# An Intelligent In-Shoe System for Gait Monitoring and Analysis with Optimized Sampling and Real-Time Visualization Capabilities

**DOI:** 10.3390/s21082869

**Published:** 2021-04-19

**Authors:** Jiaen Wu, Kiran Kuruvithadam, Alessandro Schaer, Richie Stoneham, George Chatzipirpiridis, Chris Awai Easthope, Gill Barry, James Martin, Salvador Pané, Bradley J. Nelson, Olgaç Ergeneman, Hamdi Torun

**Affiliations:** 1 Institute of Robotics and Intelligent Systems, ETH Zurich, 8092 Zurich, Switzerland; wujiae@ethz.ch (J.W.); kiranku@student.ethz.ch (K.K.); vidalp@ethz.ch (S.P.); bnelson@ethz.ch (B.J.N.); 2 Magnes AG, Selnaustrasse 5, 8001 Zurich, Switzerland; aschaer@magnes.ch (A.S.); chgeorge@magnes.ch (G.C.); oergeneman@magnes.ch (O.E.); 3 Department of Sport, Exercise and Rehabilitation, Northumbria University, Newcastle upon Tyne NE1 8ST, UK; r.stoneham@northumbria.ac.uk (R.S.); gill.barry@northumbria.ac.uk (G.B.); 4 Cereneo Foundation, Center for Interdisciplinary Research (CEFIR), 6354 Vitznau, Switzerland; chris.awai@cereneo.foundation; 5 Department of Mechanical and Construction Engineering, Northumbria University, Newcastle upon Tyne NE1 8ST, UK; james.e.martin@northumbria.ac.uk; 6 Department of Mathematics, Physics and Electrical Engineering, Northumbria University, Newcastle upon Tyne NE1 8ST, UK

**Keywords:** gait diagnosis, wearable device, graphical descriptor, real-time monitoring, telerehabilitation, digital biomarkers

## Abstract

The deterioration of gait can be used as a biomarker for ageing and neurological diseases. Continuous gait monitoring and analysis are essential for early deficit detection and personalized rehabilitation. The use of mobile and wearable inertial sensor systems for gait monitoring and analysis have been well explored with promising results in the literature. However, most of these studies focus on technologies for the assessment of gait characteristics, few of them have considered the data acquisition bandwidth of the sensing system. Inadequate sampling frequency will sacrifice signal fidelity, thus leading to an inaccurate estimation especially for spatial gait parameters. In this work, we developed an inertial sensor based in-shoe gait analysis system for real-time gait monitoring and investigated the optimal sampling frequency to capture all the information on walking patterns. An exploratory validation study was performed using an optical motion capture system on four healthy adult subjects, where each person underwent five walking sessions, giving a total of 20 sessions. Percentage mean absolute errors (MAE%) obtained in stride time, stride length, stride velocity, and cadence while walking were 1.19%, 1.68%, 2.08%, and 1.23%, respectively. In addition, an eigenanalysis based graphical descriptor from raw gait cycle signals was proposed as a new gait metric that can be quantified by principal component analysis to differentiate gait patterns, which has great potential to be used as a powerful analytical tool for gait disorder diagnostics.

## 1. Introduction

Human locomotion is one of the most important abilities that must be acquired and maintained to perform activities of daily life and, despite requiring little thought, implies a complex series of coordinated events within the body. This involves the communication of intricate sensory information, which is integrated in the nervous system and results in motor commands that control muscle activation and, finally, joint movement. Gait analysis provides important insight into the health and state of these systems. In particular, faulty sensory feedback, damaged nervous systems, or impaired muscle control can result in an alteration of normal locomotion [1,2]. Consequently, clinical gait analysis is widely used to assess the overall health status of both pediatric and adult patients [2,3]. Clinical gait analysis has also shown effectiveness in pretreatment evaluation, surgical decision making, and post-operative rehabilitation, and can also be used to recognize deterioration of walking patterns that are associated with a variety of orthopedic and neurological disorders, such as ankle sprains, rheumatoid arthritis, Parkinson’s disease, cerebral palsy, dementia, and multiple sclerosis [2,4,5,6,7,8,9].

Clinical gait analysis primarily relies on two methods, stationary, instrumented analysis and subjective qualitative observations by the physiotherapist. Each method is valuable but with limiting factors that restrict their efficacy and reliability. The first method allows for a detailed evaluation of motion, usually through either highly accurate force platforms or optical motion tracking systems. These approaches require long set-up time including marking of patients, high operational complexity due to the necessary specialized technical knowledge, and high cost for lab-based settings [10]. It is limited by spatial constraints, and the experimental equipment can be intimidating for test-subjects and thus lead to compromised observations [10]. The second method is based on physiotherapist observation, and is, therefore, limited in recognizing subtle walking pattern features and prone to subjective interpretation [3,11,12,13,14]. Under both conditions, patients walk in an idealized environment being conscious of the presence of an observer. This leads to behavior where patients either involuntarily or voluntarily focus on correcting their action [15,16]. Thus, a typical assessment session does not necessarily represent patients’ normal walking in daily life. For an ideal analysis, walking performance should not be affected by the individuals who are monitoring. Furthermore, certain gait problems (e.g., freezing [17] and spasticity [5]) happen during automatic control of gait and are typically reduced when the patient switches to goal-oriented control of gait. These instances can be difficult to detect in a clinical setting.

To address issues with current methods, researchers have analyzed many cost-effective and portable, wearable gait analysis systems [11,18,19]. These wearable sensor technologies are essential to the realization of personalized continuous gait monitoring in an unconstrained environment with minimal intervention. Typically, these sensors need to be specially mounted on the body as an additional component (e.g., attached to body [20], textile [21], or on the shoes [1,18,22,23]), which reduces wearer’s comfort and can be obtrusive and unstable. Recently, in-shoe sensing systems have been developed for gait pattern detection and pathological gait diagnosis. Zhang et al. developed a SportSole consisting of a multicell piezoresistive sensor, an inertial measurement unit (IMU), and a logic unit [24]. Pressure and acceleration data were measured to estimate the spatiotemporal gait parameters and center of pressure trajectories using a support vector regression model [24]. Carbonaro et al. used force sensor and accelerometer embedded smart shoes FootMoov to detect gait phases [25]. Nilsson et al. mounted an inertial navigation system into the shoes to estimate the walking trajectory [26]. Most of these studies focus on the assessment method of walking in healthy subjects or patients, while few existing research has considered the data acquisition bandwidth of the system for gait analysis [27]. Systems with inadequate data acquisition frequency cannot capture the walking signal in full details, such as short but fast varying movements, and lose the information at high frequency. The accuracy of the spatial gait parameter estimation will be severely affected. This is because when integrating the walking data with compromised signal fidelity, errors will also be integrated continuously, thus leading to gait parameter uncertainties and a lack of precision for gait analysis.

Characterizing human gait in a quantitative and intuitive manner has significant benefits in clinical diagnostics and rehabilitation along with improving our basic understanding of complex gait mechanisms. In spite of the rapid development of sophisticated walking data collection systems, the evaluation and communication of gait conditions remains challenging in-clinic, even for relatively common situation, such as for describing the progress of a patient’s knee recovery during rehabilitation after surgery [28]. Verbal descriptions of rehabilitation progress throughout the gait cycle tend to be imprecise. A comprehensive understanding and an objective data analysis for quantitative gait analysis is urgently needed.

In this paper, a miniaturized, low-power, cost-effective, highly mobile, and user-friendly in-shoe system embedded with inertial sensors, i.e., accelerometer, gyroscope, and magnetometer, is developed to address hardware limitations particularly related to signal bandwidth and to devise new gait metrics. The integrated in-shoe system is able to collect long-term gait data without intervention and inconvenience for the subjects in a real-world setting. The aim of this study is three-fold: (1) develop a shoe-integrated inertial sensor-based gait monitoring and analysis platform to perform reliable measurements and capture all relevant gait behaviors, investigating and optimizing system data acquisition bandwidth for gait analysis, (2) extend the well-established zero-velocity update technique with a gradient descent-based complementary Madgwick filter and heuristic techniques to identify and quantify spatiotemporal gait parameters, and (3) introduce an eigen-analysis based graphical metric quantified by principal component analysis, which can intuitively identify temporal characteristics of the gait cycles.

## 2. Materials and Methods

### 2.1. System Setup

**Figure 1:**
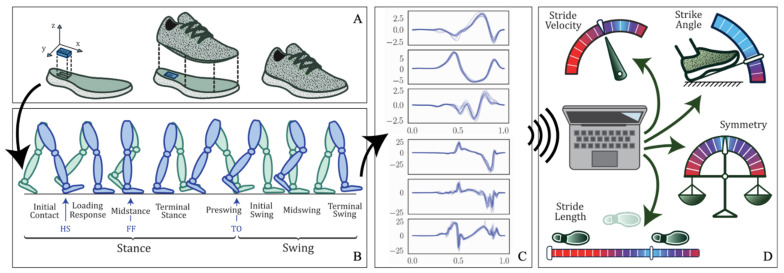
Graphical illustration of the Nushu system. (**A**) The sensor units are inserted in the outsole of the shoe; the upper part is glued so as to seal the shoe. (**B**) Gait phases during a full gait cycle. (**C**) Signal examples from the sensors. (**D**) Gait parameters generated by Nushu system.

To perform gait analysis, a pair of motion sensing shoes called Nushu is developed in this study for data acquisition. This method serves the aim of the development of a shoe-integrated gait monitoring and analysis platform referring to introduction aim (1). The system comprises a customized sensor unit inserted in the posterior portion of the outsoles of the shoes (usually thicker and less prone to bending), as shown in Figure 1A. The sensor unit is fixed in place with silicon glue, and the upper component of the shoe is glued on top with a heat-activated contact cement normally used in the shoe-manufacturing industry. This allows for a tight seal and consequently the possibility of prolonged indoor and outdoor testing.

Each sensor unit is equipped with an inductive wireless charging unit containing a rechargeable Li-Po battery of 550 mAh capacity and the receiver coil with its wireless charging circuit, a 32-bit microcontroller (ARM Cortex-M4), a Bluetooth low energy network processor (BlueNRG-MS, STMicroelectronics, Geneva, Switzerland), a micro-SD card socket, and on-board sensors (LSM6DSM and LSM303AGR, STMicroelectronics, Geneva, Switzerland), including a tri-axis accelerometer with 16-bit resolution (LSM6DSM, with selectable dynamic ranges of: ±2 g, ±4 g, ±8 g, or ±16 g), a tri-axis gyroscope with 16-bit resolution (LSM6DSM, ±125 dps, ±250 dps, ±500 dps, ±1000 dps, and ±2000 dps), and 3-axis magnetometer with 16-bit resolution (LSM303AGR, ±50 gauss). The Nushu system can record data continuously during walking as schematically illustrated in Figure 1B,C. In the meantime, the sensor data can be either logged onto a local flash memory (up to 1000 Hz) or streamed in real time via Bluetooth wirelessly to a mobile device (up to 400 Hz). To make the device user friendly, the in-shoe system can be charged wirelessly on a customized charging station. The battery can last for around eight hours when the device is logging. The developed Nushu system can be controlled by a custom designed graphical user interface (GUI) to start or stop recording, real-time monitor and visualize walking data, analyze gait data immediately after walking, and generate a feedback performance report autonomously. During walking, gait data are monitored and visualized on the mobile device in real time. After walking, the clinician can select the interested time-frame data that should be analyzed through designed GUI. Data processing on the selected walking data is performed off-line immediately. With instantly estimated spatiotemporal gait parameters, a performance feedback report containing 13 gait parameters including stride velocity, stride time, stride length, minimum foot clearance, strike angle, stance time, swing time, stance phase, swing phase, cadence, maximum angular velocity, symmetry, and variability can be generated autonomously, and displayed to the clinician immediately within 30 s (processed with Ubuntu). An example of generated performance report is shown in Supplementary Materials, and schematically presented in Figure 1D. It displays all feedback information through a graphical representation of overall walking performance.

### 2.2. Sensor Parameters Optimization

Aliasing effects have a large impact on the signal fidelity of current commercial inertial sensors. Inadequate sampling frequency can lead to critical aliasing problems, causing the digital representation of the analogue signal to be erroneous. Erroneous digital representation may not alter the estimation accuracy of the temporal parameters, but it can have a significant impact on the estimated spatial parameters, e.g., stride length and stride velocity due to the accumulation of the error caused by aliasing effect during the integration process. Therefore, optimizing the sampling frequency of the sensor is critical for improving their performance and also increasing the accuracy of the gait analysis results. This method serves the aim of investigating and optimizing the system data acquisition bandwidth for gait analysis, referring to the aim (1) mentioned in the introduction.

**Figure 2:**
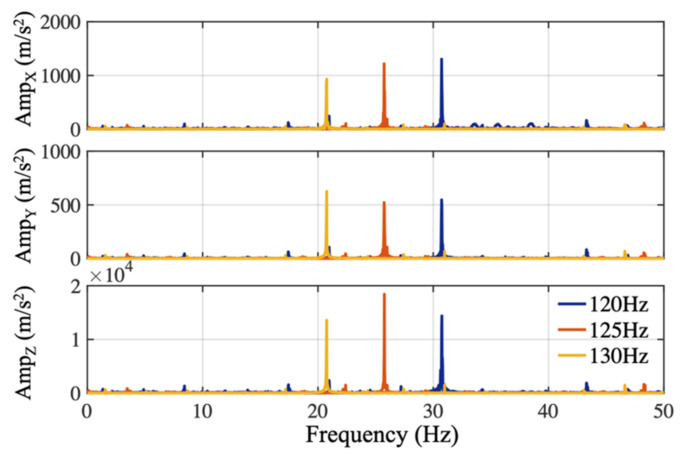
Aliasing frequency signals resulting from sampling 120, 125, and 130 Hz audio waves at 100 Hz.

An antialiasing filter reduces undesired-frequency components above the Nyquist frequency prior to digital sampling. The antialiasing filter characteristics of the IMU sensor (BNO055, Bosch, which was embedded in shoes in the preliminary stage) was experimentally obtained in a setup where the sensor configured to a sample rate of 100 Hz was fixed on the surface of a speaker. Sinusoidal audio waves spanning a duration of one minute with different frequencies of 120 Hz, 125 Hz, and 130 Hz, which were slightly above the IMU sampling frequency, were generated using a computer (using Matlab R2017a, Mathworks). Those audio waves were played through the speaker and recorded by the accelerometer of the IMU sensor. The digitally sampled accelerometer data recorded in the time-domain are mathematically transformed into the frequency domain by the fast Fourier transform (FFT) for spectral analysis shown in Figure 2. The actual IMU digitized the data at around 99 Hz, low frequency components at 21 Hz, 26 Hz, and 31 Hz were observed on the spectrum, illustrating the absence of the appropriate anti-aliasing filter before analogue to digital conversion in this commercialized IMU sensor.

Moreover, even if the antialiasing filtering is present in IMU sensors, valuable high-frequency information above the cut-off frequency of antialiasing filter, such as fast variations in the acceleration, could still be lost [27]. Therefore, knowing the highest frequency component within a walking time series in advance can help deciding the optimal sampling rate, which is sufficient to precisely estimate gait parameters, but not too high so that the system remains power and memory efficient [29].

Nushu system was evaluated against two different sensors to determine the optimal sampling frequency and to investigate the validity of the choice of 100 Hz as the sampling frequency, which is a common choice for gait analysis [21,30,31,32]. The first sensor is a wired, high-sensitivity piezoelectric accelerometer sensor (AT/14, DJB Instruments, Suffolk, UK). It can sample up to 5000 Hz with an antialiasing filter applied priori. The second sensor is a wireless commercial sensor (AX3, Axivity Ltd, Newcastle upon Tyne, UK) with only an accelerometer and a maximum sampling rate of 100 Hz, with no antialiasing filter priori. Nushu system, which has both accelerometers and gyroscopes was set to sample at 100 Hz, with no antialiasing filter priori during these measurements. All three sensors were attached to a healthy subject’s instep position of the shoes using straps. This healthy subject has no pathologies that affect gait. The subject was asked to walk in a straight line about 6 m and walk back. Three collected sets of time series acceleration data were normalized individually after removing DC bias (by means of selecting a short segment (5 s) of the stationary signals, and subtracting the mean of the stationary signals). Each set of the obtained normalized acceleration data is denoted as $\left\{{\hat{a}}_{ix}\left(k\Delta t_{i}\right),\;{\hat{a}}_{iy}\left(k\Delta t_{i}\right),\;{\hat{a}}_{iz}\left(k\Delta t_{i}\right)\right\}$, where $\Delta t_{i}=\frac{1}{f_{i}}$, $f_{i}$ is the sampling frequency, $k\in {Ν}^{+}$, and $i\in \left\{1,\;2,\;3\right\}$ represents for three different sensors. Since no clock was shared between those three acquisition systems, a synchronization process was implemented as follows. The magnitude of acceleration was first calculated for each set as

(1) $${\hat{a}}_{iM}\left(k\Delta t_{i}\right)=\sqrt{\sum_{j\in \left\{x,\;y,\;z\right\}}{\hat{a}}_{ij}^{2}\left(k\Delta t_{i}\right)}.$$

Then an antialiasing filter was applied to ${\hat{a}}_{iM}\left(k\Delta t_{i}\right)$ and down-sampled ${\hat{a}}_{iM}\left(k\Delta t_{i}\right)$ to $\min_{i}f_{i}$ such that all signals have the same time interval Δt. Time series ${\hat{a}}_{1M}\left(k\Delta t\right)$ and ${\hat{a}}_{2M}\left(k\Delta t\right)$ were shifted along the time axis such that their cross-correlation with ${\hat{a}}_{3M}\left(k\Delta t\right)$ was maximized. According to the obtained time lag based on the maximum correlation time point, the raw time series data $\left\{a_{ix}\left(k\Delta t_{i}\right),\;a_{iy}\left(k\Delta t_{i}\right),\;a_{iz}\left(k\Delta t_{i}\right)\right\}$ were shifted along their time axes for synchronization. Using the synchronized data, each walking stride was detected and separated for spectral analysis with the help of the gyroscope from Nushu inertial sensor.

Furthermore, to find the optimal sampling rate for gait analysis, the signals with the highest sampling frequency (5000 Hz) from the piezoelectric sensor were cut off by low pass filters at different cut-off frequencies. The percent root mean squared errors (RMSE (%)) between the filtered data and the original data were compared after applying different cut-off frequencies. Then the optimal sampling rate was determined by balancing the RMSE (%) and the cut-off frequency.

### 2.3. Gait Analysis Method

In this section, the method of quantifying spatiotemporal gait parameters, which serves the aim (2) referred in the introduction, was introduced. To assess the gait performance, each gait cycle was divided into multiple gait phases such that quantitative gait features, including temporal and spatial gait parameters, can be evaluated. The gait cycle was typically divided into two main phases by gait events, stance and swing, as shown in Figure 1B. Those two phases can further be subdivided into eight functional phases, five during stance and three during swing. The first two phases, initial contact and loading response, occur during the weight acceptance with double feet support. Mid-stance and terminal stance go on during the single foot support, followed by the preswing phase where the forward limb motion starts. Afterwards, the swing phase commences with the initial swing, in which the hip and knee start to bend in tandem with ankle dorsiflexion. The mid-swing immediately follows when the swinging leg is aligned with the standing leg. Finally, the terminal swing occurs when the leg decelerates by contraction of the hamstrings and prepares for ground contact [3,33]. The three key gait events to anchor these phases are heel strike (HS), when the heel strikes the ground at initial contact, flat foot (FF), when the foot is flat on the ground, and toe off (TO), when the toes leave the ground.

With the defined gait phases, 13 gait metrics are configured to be estimated by the Nushu system, as listed in Table 1. Additional gait parameters can be customized based on user requirements.

Given the nature of the gait metrics, raw kinematic data from the inertial sensors are first transferred into the global reference frame to detect characteristic events during the walking cycle and estimate the orientation of the sensors. This allows for the extraction of spatial features through integration of the measured accelerations and angular velocities.

#### 2.3.1. Attitude Estimation and Heuristic Techniques

The orientation of the foot is estimated through sensor fusion, which is a technique often used for attitude and heading reference systems (AHRS) that makes use of gyroscope, accelerometer, and magnetometer measurements through specific filters. Among many that have been implemented in previous work, our system uses the algorithm derived by Madgwick [34]. This gradient descent based complementary filter employs a quaternion representation and uses the gravitational acceleration and the Earth’s magnetic field picked up by the accelerometer and magnetometer to estimate the orientation error that arises from naively integrating the raw angular data from the gyroscope. Compared to other filters, e.g., Kalman or extended Kalman filters, Madgwick’s algorithm compensates the magnetic distortion to eliminate the need for predefining the reference magnetic field direction [34]. It is also computationally less expensive and offers the potential of real-time data processing on Nushu’s microcontroller.

If the sensor moves arbitrarily in space, the gravitational acceleration will become mixed with other linear accelerations. Heuristic techniques are required for the correct implementation of the Madgwick filter and the post-processing algorithm. These techniques exploit the fact that gait is a periodic series of alternate stances and swings. It is widely accepted that during the stance phase there is a short period of time, referred to as mid-stance, where the foot is stationary, while the shank pivots around the ankle, leading to linear accelerations of zero on the foot. Assuming that during mid-stance, the accelerometer measures only the gravitational acceleration, these stationary periods are used as flags for the AHRS algorithm.

**Figure 3:**
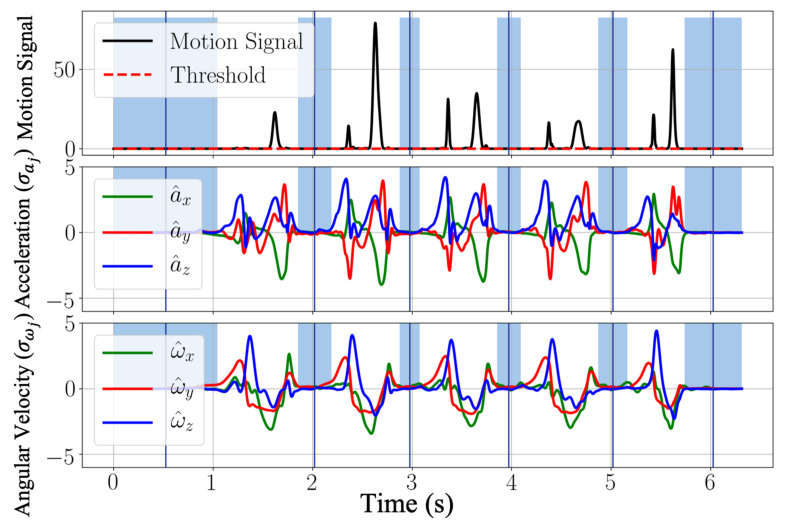
Example signals of motion signals, normalized acceleration, and angular velocity. The threshold for the recognition of the stationary events is calculated by considering the magnitude of the motion signals. The stationary regions are highlighted in blue.

Here, we introduced an approach to detecting dynamic phases and stationary phases by the following method. We defined a new motion signal to be

(2) $$\mathrm{MS}\left(k\right)=\left|\prod_{j\in \left\{x,\;y,\;z\right\}}{\hat{a}}_{j}\left(k\right){\hat{\omega }}_{j}\left(k\right)\right|,$$

where ${\hat{a}}_{j}\left(k\right)$ and ${\hat{\omega }}_{j}\left(k\right)$ represent the normalized acceleration (${\hat{a}}_{j}\left(k\right)=\frac{a_{j}\left(k\right)-{\bar{a}}_{j}}{\sigma_{a_{j}}}$) and angular velocity (${\hat{\omega }}_{j}\left(k\right)=\frac{\omega_{j}\left(k\right)-{\bar{\omega }}_{j}}{\sigma_{\omega_{j}}}$) with the mean ${\bar{a}}_{j}$, ${\bar{\omega }}_{j}$, and the standard deviation $\sigma_{a_{j}}$, $\sigma_{\omega_{j}}$. The motion signal is further filtered by a moving-average filter $f_{ma}\left(\mathrm{MS},\;N\right)$, where N=5 is the moving window size. Then by comparing the filtered motion signal (${\mathrm{MS}}_{f}\left(k\right)$) and an empirically determined threshold ($T_{\mathrm{MS}}=10^{-6}$), dynamic phases and stationary phases are differentiated by

(3) $$F\left(k\right)=\left\{\begin{array}{cc} 1, & {\mathrm{if}\;\mathrm{MS}}_{f}\left(k\right)<T_{\mathrm{MS}}\\ 0, & \mathrm{else} \end{array}\right.$$

where $F\left(k\right)$ is the binarized flag. The dynamic and stationary phase differentiation result is shown in Figure 3. The blue regions depict stationary phases, and the white regions represent the dynamic phases.

**Figure 4:**
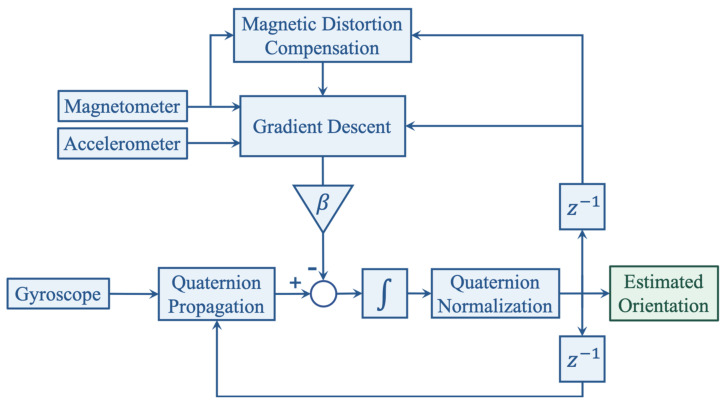
Block diagram of the orientation estimation through sensor fusion [34].

**Figure 5:**
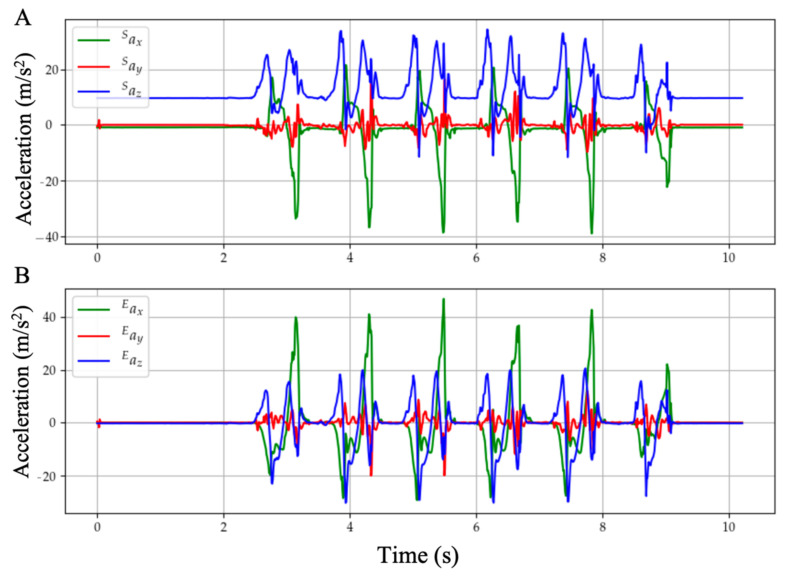
(**A**) Raw acceleration data measured in the sensor’s coordinate frame. By observing the initial stationary region, it can be deduced that the sensor’s resting position is not perfectly aligned with the ground (gravity has a slight projection onto the x and y axes). Gravity is affecting the readings of the linear accelerations and since the sensors are changing orientation it cannot be subtracted. (**B**) Transformed accelerations in the global coordinate frame. Since gravity is only projected in the global z axis, it can be directly subtracted.

With these stationary flags, the orientation of the global frame relative to the sensor frame is estimated through sensor fusion by Madgwick’s filter, as shown in Figure 4. The filter gain β, which represents gyroscope measurement error, in gradient descent is calibrated as 0.1. The acceleration data can thus be transformed from sensor frame into the global reference frame, as shown in Figure 5. Afterwards, each gait cycle is collected by segmenting the midpoint of the stationary regions for further processing.

#### 2.3.2. Event Detection Algorithm

**Figure 6:**
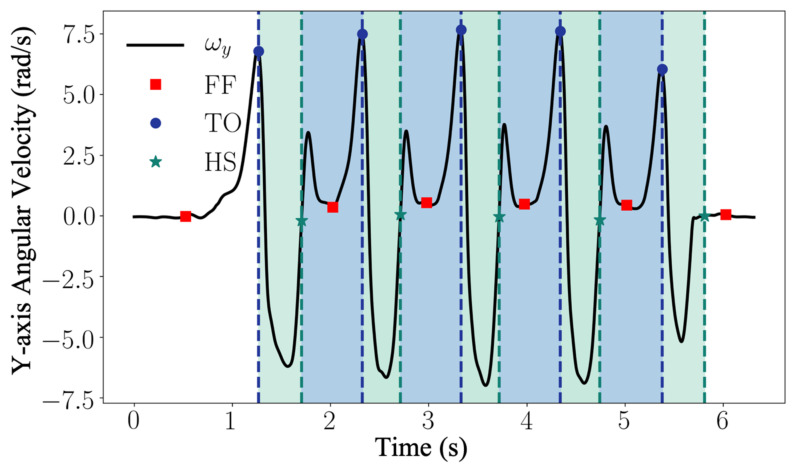
The signal from the gyroscope aligned with the medio-lateral axis of the foot is used for gait event detection. Each stride is characterized by a sequence of FF, TO, HS, and FF as indicated. The green and blue areas refer to the swing and stance phases. The blue and green vertical dashed lines define the starting time frame of the swing and stance phases, respectively.

During a gait cycle, the rotation of the foot around the mediolateral axis (flexion/extension axis of the ankle) is the most prominent movement comparing to supination/pronation and inversion/eversion. Therefore, the key gait events HS, FF, and TO can be detected by inspecting the angular velocity aligned with the mediolateral axis of the foot ($\omega_{y}$) [30]. For a normal gait cycle, the foot rotation around the mediolateral axis changes as follows: a gait cycle begins with the foot flat on the ground; the foot starts to rotate forward with the toe as the contact point, the heel leaves the ground first with the rotation speeding up; then the toe leaves the ground, the rotation slows down; as the foot leans forward, the rotation reverses; near the end of the foot forward movement, the toe elevation exceeds the heel elevation, the rotation reverses again after the heel strike the ground; in the end of the gait cycle, the toe lower down with the heel as the contact point until the foot is flat again [30]. In Figure 6, the low pass filtered normal walking signal ${\overset{ˇ}{\omega }}_{y}=f_{L}\left(\omega_{y},\;10\right)$ from the gyroscope y axis is presented. The signals of walking cycles display a characteristic pattern with well-defined features (peaks and plateaus) that are associated with the gait events. The rules for event detection from the angular velocity ${\overset{ˇ}{\omega }}_{y}$ are predefined according to the ground truth provided by the motion capture system. The detection algorithm uses a set of dynamic thresholds and local peak-identification techniques to recognize the sequence of the events. In particular, for each dynamic motion region segmented with Equation (3), the TO is associated with the strongest local maximum within the filtered cycle signal ${\overset{ˇ}{\omega }}_{y}^{n}\left(k\right)$, where *n* is the *n*th segment of dynamic region. The time instance of TO can be found as the time index $k_{\mathrm{TO}}^{n}$, which satisfies the following conditions:

(4) $${\overset{ˇ}{\omega }}_{y}^{n}\left(k_{\mathrm{TO}}^{n}\right)>std\left({\overset{ˇ}{\omega }}_{y}^{n}\right),\;{\overset{ˇ}{\omega }}_{y}^{n}\left(k_{\mathrm{TO}}^{n}\right)>{\overset{ˇ}{\omega }}_{y}^{n}\left(k_{\mathrm{TO}}^{n}-1\right)\;\mathrm{and}\;{\overset{ˇ}{\omega }}_{y}^{n}\left(k_{\mathrm{TO}}^{n}\right)>{\overset{ˇ}{\omega }}_{y}^{n}\left(k_{\mathrm{TO}}^{n}+1\right).$$

The HS corresponds to the following rising zero-crossing within the updated dynamic region. The time instance of HS can be found as the time index $k_{\mathrm{HS}}^{n}\;$, which satisfies the conditions as follows:

(5) $${\overset{ˇ}{\omega }}_{y}^{n}\left(k_{\mathrm{HS}}^{n}-1\right)\cdot {\overset{ˇ}{\omega }}_{y}^{n}\left(k_{\mathrm{HS}}^{n}\right)<0\;\mathrm{and}\;{\overset{ˇ}{\omega }}_{y}^{n}\left(k_{\mathrm{HS}}^{n}\right)>{\overset{ˇ}{\omega }}_{y}^{n}\left(k_{\mathrm{HS}}^{n}-1\right).$$

The FF event is observed as the first local minimum between heel strike and toe off. The time instant of FF can be found as the time index $k_{\mathrm{FF}}^{n}\;$ satisfying the following conditions:

(6) $${\overset{ˇ}{\omega }}_{y}^{n}\left(k_{\mathrm{FF}}^{n}\right)<{\mathrm{ths}}_{\mathrm{FF}},\;{\overset{ˇ}{\omega }}_{y}^{n}\left(k_{\mathrm{FF}}^{n}\right)<{\overset{ˇ}{\omega }}_{y}^{n}\left(k_{\mathrm{FF}}^{n}-1\right)\;\mathrm{and}\;{\overset{ˇ}{\omega }}_{y}^{n}\left(k_{\mathrm{FF}}^{n}\right)<{\overset{ˇ}{\omega }}_{y}^{n}\left(k_{\mathrm{FF}}^{n}+1\right),$$

where ${\mathrm{ths}}_{\mathrm{FF}}$ is the empirical dynamic threshold defined as $\mathrm{avg}\left({\overset{ˇ}{\omega }}_{y}^{n}\right)+0.1\cdot \mathrm{std}\left({\overset{ˇ}{\omega }}_{y}^{n}\right)$. This instant resides within a plateau region of the gyroscope signal and generally represents the angular velocity, which is closest to 0 dps.

In order to further process the data, only valid strides (which contain the correct sequence <FF-TO-HS-FF>) have been considered. Furthermore, the event detection algorithm was used to extract temporal parameters such as swing time, stance time, swing phase, and stance phase (as described in Table 1). Stance time was calculated as the time interval between HS and the next TO, swing time was calculated as the time interval between TO and the next HS. Stance and swing phases were calculated as the ratio with respect to a full stride time.

#### 2.3.3. Spatio-Temporal Parameters Estimation

Drift accumulates due to the numerical integration of the acceleration errors. This is addressed by using the zero-velocity update technique (ZUPT), whose main assumptions are that velocities and displacement are equal to zero during mid-stances, and that integration drift within a gait cycle is accumulated linearly [35,36]. Therefore, numerical integration can be carried out piecewise at every gait-cycle (using stationary intervals as reset points) to avoid the propagation of drift throughout the signal, and linear dedrifting is applied to each cycle to remove drift and discontinuities as shown in Equations (8) and (10). Through the implementation of the AHRS and ZUPT algorithms, the accelerations are integrated in the global reference frame to extract spatial features such as stride velocity and stride length as follows:

(7) vjnk=vjnk−1+a Ejnk·Δt,

(8) v djnk=vjnk+kTnvjn0−vjnend,

(9) sjnk=sjnk−1+v djnk·Δt,

(10) s djnk=sjnk+kTnsjn0−sjnend,

where $j\in \left\{x,\;y,\;z\right\}$, $v_{j}^{n}$ and $s_{j}^{n}$ are the velocity and displacement of the *n*th valid stride motion region, v djn and s djn are the dedrifted velocity and displacement of *n*th valid stride, $v_{j}^{n}\left(0\right)$ and $s_{j}^{n}\left(0\right)$ is the initial velocity (equal to zero) of *n*th valid stride, $v_{j}^{n}\left(end\right)$ and $s_{j}^{n}\left(end\right)$ are the velocity and the displacement at the end of the *n*th valid stride and $T^{n}$ is the number of the samples in the *n*th valid stride. Gait speed is calculated as the mean in-plane velocity across a full gait stride:

(11) vn=1Tn∑k=0k=endv dxnk2+v dynk2,

where $v^{n}$ is the stride speed of the *n*th valid stride. Stride length is expressed as:

(12) sn=∑k=0k=ends dxnk2+s dynk2, 

Cadence is expressed as a function of stride length and stride speed:

(13) $$\mathrm{cadence}=60\cdot \frac{v^{n}}{s^{n}}\cdot 2,$$

The symmetry ratio (SR) of a particular parameter is calculated as

(14) $$\mathrm{SR}=\frac{L-R}{\frac{L+R}{2}}\;,$$

where L and R are the value of the considered parameter for the left and right foot. The sign of the SR indicates the bias direction, where a positive SR indicated a left bias whilst a negative SR corresponds to a right bias. The gait variability of a particular parameter can be calculated as:

(15) $$var=\frac{\mu }{\sigma \;},\;$$

where μ and σ is the mean and standard deviation value of the considered parameter, respectively.

**Figure 7:**
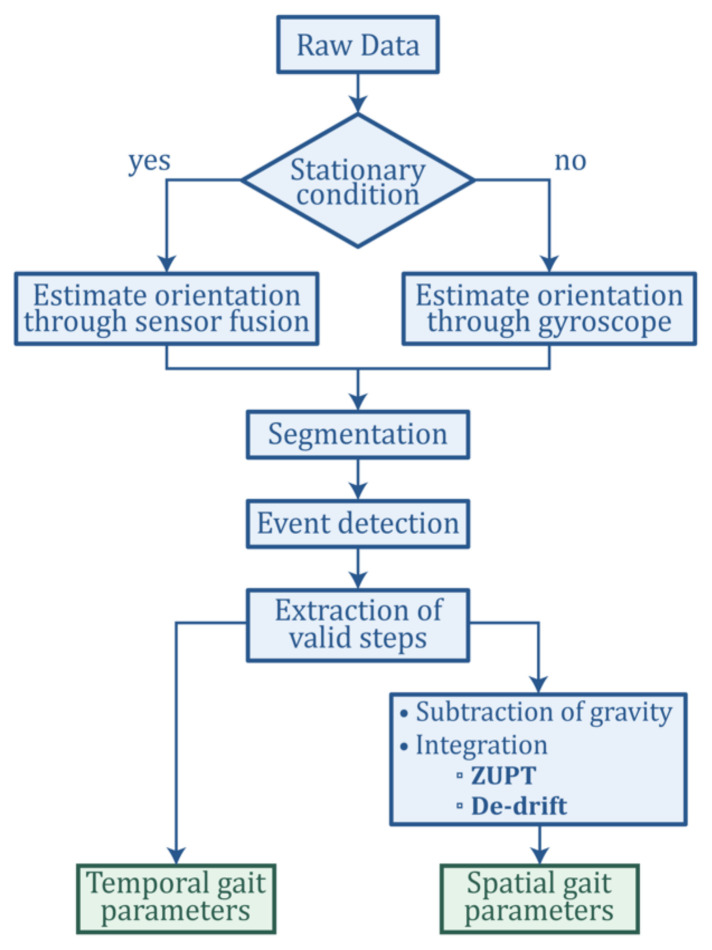
The flowchart of the data processing procedure.

The gait analysis procedure discussed in this section is summarized in Figure 7.

### 2.4. Validation Method

**Figure 8:**
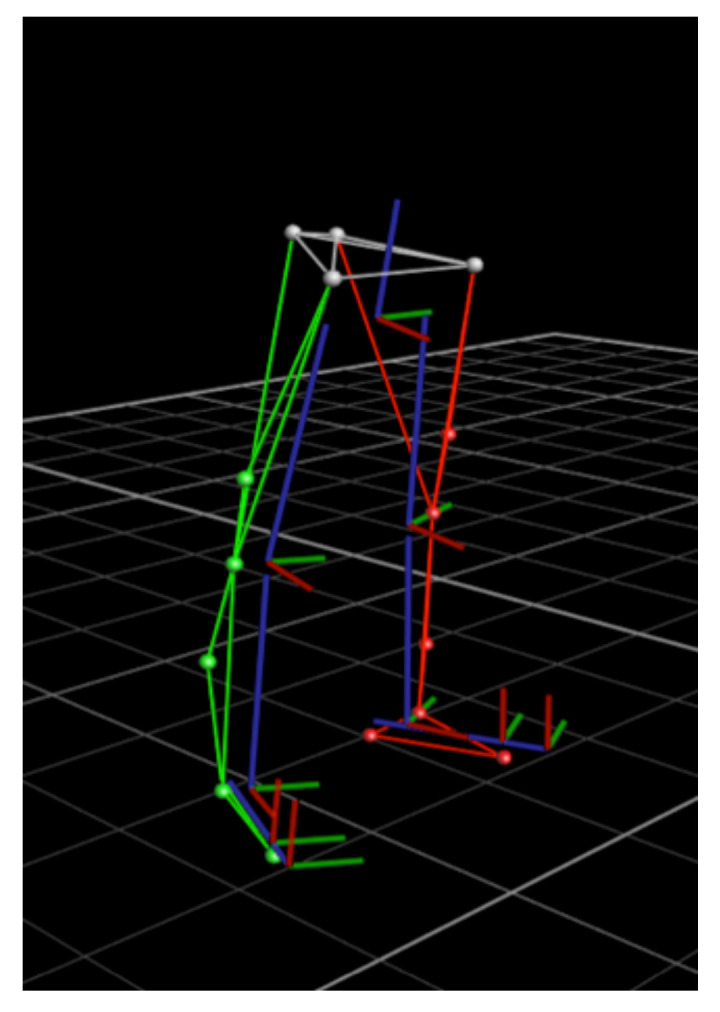
Subject lower limb skeleton model from 3DGA markers.

To evaluate whether gait parameters could be accurately estimated by Nushu, four healthy adults (with subjects’ information shown in Table 2), each perform five walking sessions, twenty trials in total were measured and their data compared between optical motion capture system and Nushu in a validation experiment. Sixteen reflective markers were attached to each subject’s shoes and lower limbs, i.e., anterior superior iliac spine (ASIS), posterior iliac spine (PSIS), distal lateral thigh, lateral femur epicondyle, distal lateral shank, lateral malleoli, above the toe (second metatarsal phalangeal joint) and behind the calcaneus (the same height as the toe marker). This is a standard lower limb gait marker set. A highly accurate optical 3D gait analysis (3DGA) system (Vicon Oxford Metrics, Ltd., Oxford, UK) with fourteen infrared cameras, which served as the reference system, was used to track the instantaneous position of markers located on subject’s body segments during the walking experiment. The lower limb skeleton model constructed from 3DGA markers is presented in Figure 8. Both the optical motion capture system and the optimized Nushu system recorded walking data simultaneously at the optimal frequency $f_{opt}$, which is determined by the sampling optimization described in Section 2.1.

After the system set up, each healthy adult subject was instructed to complete five walking sessions along the same 10-m long, straight line route, which was marked on the floor with yellow tape. During 20 walking sessions, each subject stood stationary for five seconds and then walked to the end of the marked area at his/her comfortable speed under the guidance of a computer-generated voice. The Nushu system was reset between each subject’s walking test. Each walking session was also video recorded for later analysis in the case of unexpected events. After experiments, the position of calcaneus marker, which is closest to the sensor placement, was used to detect the heel strike and estimate the foot velocity and the displacement. The marker above the second metatarsal bone was used to detect the toe off. The velocity was estimated by differentiating the calcaneus marker locations as

(16) $$v_{\mathrm{Vicon}}=\;{\dot{p}}_{\mathrm{Vicon}},$$

where $p_{\mathrm{Vicon}}$ is the position of 3DGA markers. The stride length was calculated as

(17) $$L_{\mathrm{Vicon}}\left(k\right)=p_{\mathrm{HS}}\left(k\right)-p_{\mathrm{HS}}\left(k-1\right),\;k\in {Ν}^{+}.$$

To access if significant statistical differences in spatiotemporal gait parameters exist between the Nushu and 3DGA systems, independent *t*-tests were investigated to compare the two acquisition systems. The critical p value was set to α=0.05.

### 2.5. A New Gait Metric

To characterize human gait in a simple and intuitive way, a new gait hodograph descriptor obtained from the geometric gait features, was proposed in this section, which serves the aim (3) as stated in the introduction. This gait descriptor reflects the characteristics of gait patterns without complex calculations and detects abnormal gait patterns intuitively [37]. This is in contrast to advanced gait analysis algorithms, which can achieve relatively high performance, but are computationally expensive and cannot avoid errors in estimating the quantitative gait metrics.

For each gait cycle, a gait hodograph is formed by neglecting the time dimension and directly plotting the low pass filtered motion data from the accelerometer and gyroscope sensors. The low pass filter with a cut off frequency 20 Hz is applied to get the main characteristics, such that the shape characteristic is not noisy, but still contains the dominant information in the low frequencies. Among all motion signals obtained from the triaxial accelerometer, the triaxial gyroscope and the triaxial magnetometer, the trajectories of three most prominent signals $\left\{a_{x}\left(t\right),\;a_{z}\left(t\right),\;\omega_{y}\left(t\right)\right\}$ from each person’s left and right foot are simultaneously reconstructed into 2-D planar projections, $\left\{a_{x}\left(t\right),\;\omega_{y}\left(t\right)\right\}$, $\left\{a_{x}\left(t\right),\;a_{z}\left(t\right)\right\}$, $\left\{a_{z}\left(t\right),\;\omega_{y}\left(t\right)\right\}$.

To quantitatively differentiate different people’s gait type by those secondary signals $\left\{a_{x}\left(t\right),\;a_{y}\left(t\right)\right\}$, $\left\{\omega_{x}\left(t\right),\;\omega_{z}\left(t\right)\right\}$, principal component analysis (PCA) is employed to represent and distinguish characteristics of the gait features. PCA is a powerful analysis tool for identifying data patterns and representing data sets to highlight their differences and similarities with minimum information loss. In order to implement PCA analysis, $\left\{a_{x}\left(t\right),\;a_{y}\left(t\right)\right\}$, $\left\{\omega_{x}\left(t\right),\;\omega_{z}\left(t\right)\right\}$ signals of each step cycle were resampled to 251×4 data points. Then, the median step cycle was selected among the time series to represent each person’s gait and then normalized for a detailed analysis. This normalization refers to mean centering, subtracting the average values from each cycle data of median step to make its empirical mean zero. The resultant mean centering data were denoted by $\left\{{\tilde{a}}_{x}\left(k\right),\;{\tilde{a}}_{y}\left(k\right)\right\}$, $\left\{{\tilde{\omega }}_{x}\left(k\right),\;{\tilde{\omega }}_{z}\left(k\right)\right\}$, where $k\in \left\{1,\;2,\;\ldots 251\right\},$ and then were formulated into a matrix form as

(18) $$\tilde{A}=\left[\begin{array}{cc} {\tilde{a}}_{x}\left(1\right) & {\tilde{a}}_{y}\left(1\right)\\ \vdots & \vdots \\ {\tilde{a}}_{x}\left(251\right) & {\tilde{a}}_{y}\left(251\right) \end{array}\right],\;\tilde{W}=\left[\begin{array}{cc} {\tilde{\omega }}_{x}\left(1\right) & {\tilde{\omega }}_{z}\left(1\right)\\ \vdots & \vdots \\ {\tilde{\omega }}_{x}\left(251\right) & {\tilde{\omega }}_{z}\left(251\right) \end{array}\right]$$

Afterwards, PCA was performed on these matrices.

## 3. Results

### 3.1. Sampling Frequency Optimization

**Figure 9:**
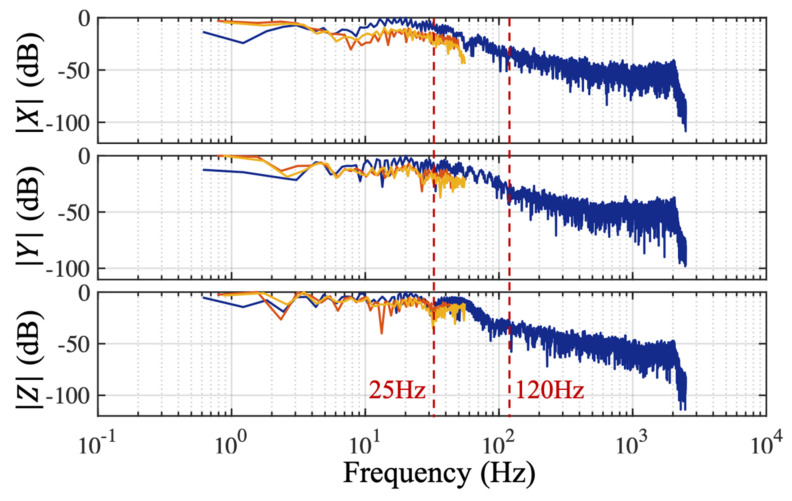
Spectrum of acceleration signals from one normalized stride sampled at 5000 Hz (blue: DJB), 100 Hz (red: Nushu), and 100 Hz (yellow: Axivity).

**Figure 10:**
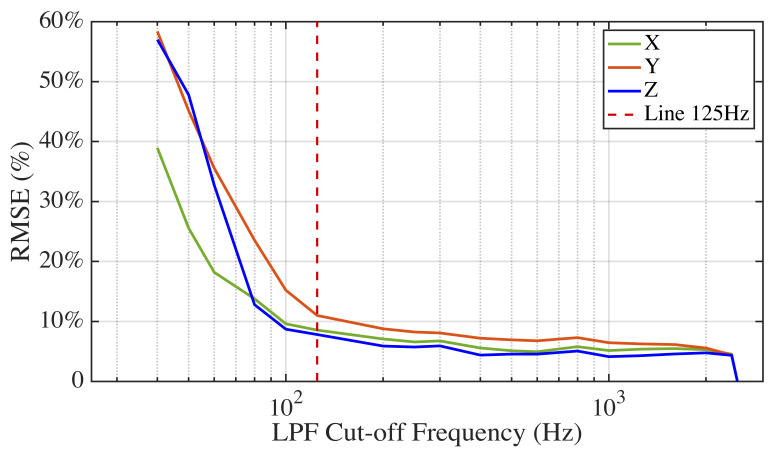
RMSE (%) between low-pass filtered signals and raw signals for a normalized stride as a function of LPF cut-off frequency.

In Figure 9, a spectrum of acceleration signals from one normalized average stride captured by three different sensors sampled at 5000 Hz (blue: DJB), 100 Hz (red: *Nushu*), and 100 Hz (yellow: Axivity) is presented. The amplitude indicates the strength of the frequency components relative to noise. We can see that the amplitude flatness was maintained up to 25 Hz for all X, Y, and Z components. Subsequently, amplitudes started to decline until approximately 120 Hz, while they were still significantly larger than the amplitude of the high-frequency noise, which indicates that there were useful frequency components between 25 and 120 Hz. Two sensors sampled at 100 Hz could not capture useful information between 50 and 120 Hz as shown in Figure 9. This shows that a sampling rate of 100 Hz was not adequate to capture all the relevant information for gait analysis. To find the optimal sampling frequency for gait analysis, the normalized detected stride data from the highest resolution piezoelectric sensor data was first processed with a series of low pass filters (LPFs). Those LPFs have stopband frequencies ranging from 50 to 2500 Hz. The RMSE (%) between raw data and filtered data of the X, Y, and Z axis as a function of LPF cut-off frequency is shown in Figure 10. As the LPF cut-off frequency increased from 25 to 125 Hz, the RMSE (%) decreased significantly. However, the further increase of LPF cut-off frequency above 125 Hz had no significant effect on the RMSE (%). Based on the trade-off between RMSE (%) and LPF cut-off frequency, the sampling frequency 250 Hz (twice of the stride frequency 125 Hz) was chosen as the optimal frequency $f_{opt}$, which could not only capture the most stride information but also save energy and memory. This result also shows a good agreement with the study in [27], which has found that the lowest sampling frequency for gait analysis lies between 200 and 300 Hz. Those results are in line with the aim (1) mentioned in the introduction.

### 3.2. Performance Evaluation

**Figure 11:**
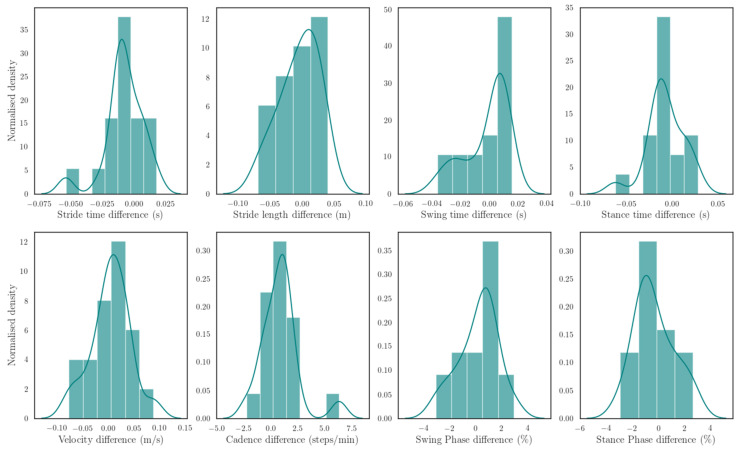
Histogram of error distribution of eight parameters for validation: stride time, stride length, swing time, stance time, velocity, cadence, swing phase, and stance phase.

Eight parameters, stride time, stride length, swing time, stance time, velocity, cadence, swing phase, and stance phase, were calculated using Nushu and compared to the ones calculated based on reference data from 3DGA. For each stride, the differences between Nushu and 3DGA of these eight parameters across all subjects’ strides were shown in the normalized histogram (Figure 11). The error metrics, mean absolute error (MAE), MAE (%), RMSE, and MAE standard deviation (SD), averaged by each session for all subjects, and *p* values are reported in Table 3. Percentage MAEs for stride time, stride length, stride velocity, cadence, swing time, stance time, swing phase, and stance phase were 1.19%, 1.68%, 2.08%, 1.23%, 3.02%, 2.59%, 3.2%, and 2.12%, respectively. Bold p values, where p<0.05, indicate significant differences between Nushu and 3DGA systems.

### 3.3. A New Gait Metric

**Figure 12:**
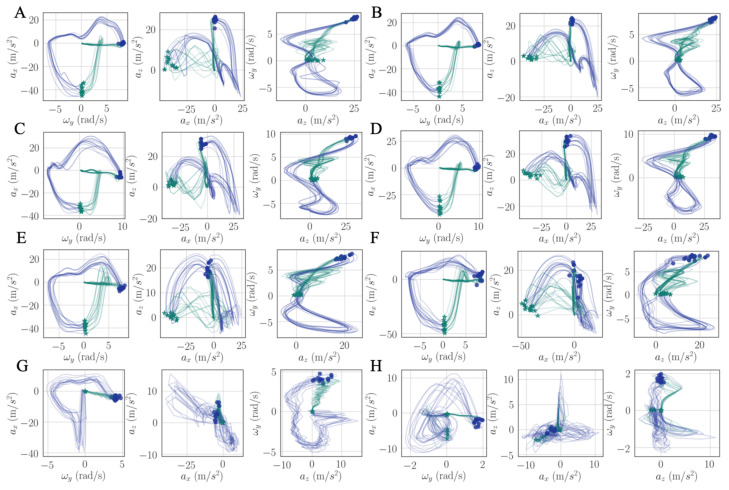
Hodographs of prominent walking signals $\left\{a_{x}\left(t\right),\;\omega_{y}\left(t\right)\right\}$, $\left\{a_{x}\left(t\right),\;a_{z}\left(t\right)\right\}$, $\left\{a_{z}\left(t\right),\;\omega_{y}\left(t\right)\right\},$ gait cycle trajectories for three healthy persons (left/right: **A**/**B**, **C**/**D**, **E**/**F**), and a stroke patient (less impaired/more impaired: **G**/**H**). Green star markers: HSs, blue circle markers: TOs, green solid lines: stance phases, blue solid lines: swing phases.

In this section, gait hodographs were analyzed based on the methods explained in Section 2.4. The results serve the aim (3) stated in the introduction. Figure 12 shows 2-D planar projections of walking cycle trajectories from three healthy persons’ left (Figure 12A,C,E) and right foot (Figure 12B,D,F), and one stroke patient’s (recording performed in a different experiment) left (Figure 12G) and right (Figure 12H) foot. The stroke patient’s walking data on a treadmill are collected by Nushu in this section only for metric comparison. The corresponding walking movements are shown in Supplementary Materials Video S1–S3 for healthy persons and Video 4 for stroke patient. To eliminate the noisy vibration from the treadmill, the collected data were filtered with 20 Hz low pass filter to get a main graphical characteristic. In these hodographs, the closed planar shape is encircled once in the counterclockwise direction for each gait cycle. Green stars mark the HS, blue circles mark the TO. The green lines represent stance phases, blue lines describe swing phases. Subtle differences of shapes and positions are observed among three different healthy persons, for example, the variability in gait cycles of the first person (Figure 12A,B) was smaller than the other two healthy persons (Figure 12C–F). However, all hodographs of healthy individuals had the same well-recognizable shape characteristics. From the stroke patient’s hodograph (Figure 12G,H), the gait patterns of both feet significantly differed from healthy individuals. Recognizable shape characteristic for the right foot were not apparent. In addition, the range of $a_{x}\left(t\right),\;a_{z}\left(t\right),\;\mathrm{and}\;\omega_{y}\left(t\right)$ for the stroke patient’s right foot was significantly smaller than the ones for healthy individuals, which reflected reduced flexibility of the ankle dorsiflexion and knee flexion caused by stroke. This also agreed well with the fact that the stroke affected this patient’s right body more severely than the left side.

**Figure 13:**
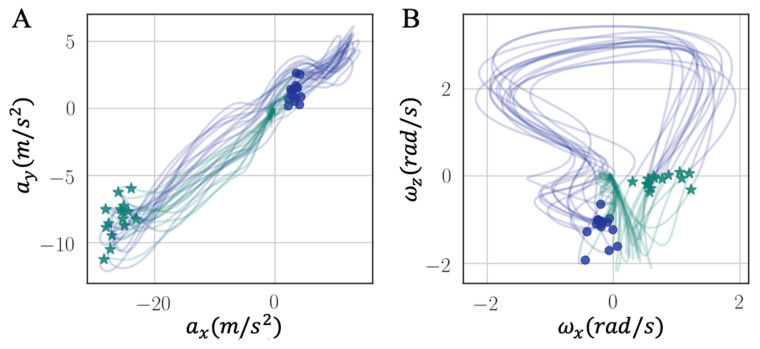
Planar 2-D projection of secondary signals (**A**) $\left\{a_{x}\left(t\right),\;a_{y}\left(t\right)\right\}\;$ and (**B**) $\left\{\omega_{x}\left(t\right),\;\omega_{z}\left(t\right)\right\}$ from a healthy subject. Green star markers: HS. Blue circle markers: TO. Green line: stance phase. Blue line: swing phase.

In Figure 13, a planar 2-D projection of $\left\{a_{x}\left(t\right),\;a_{y}\left(t\right)\right\}$ and $\left\{\omega_{x}\left(t\right),\;\omega_{z}\left(t\right)\right\}$ are presented to provide information about foot movement forward-and-backward, left-and-right in the horizontal plane, and the rotation of the ankle joints around the raw and yaw axis but does not indicate up-and-down foot movement and rotation around the pitch axis during walking. When healthy people walk along a straight path, the feet did not significantly sway from side to side. Therefore, $a_{y}\left(t\right)$ was limited to a small range. The 2-D projection hodograph of $\left\{a_{x}\left(t\right),\;a_{y}\left(t\right)\right\}$ should be in an elongated ellipsoid. For the projection of $\left\{\omega_{x}\left(t\right),\;\omega_{z}\left(t\right)\right\}$, the ankle joint’s rotation around raw and yaw axes was limited, and the signal should be dispersed around the original point of the coordinate.

**Figure 14:**
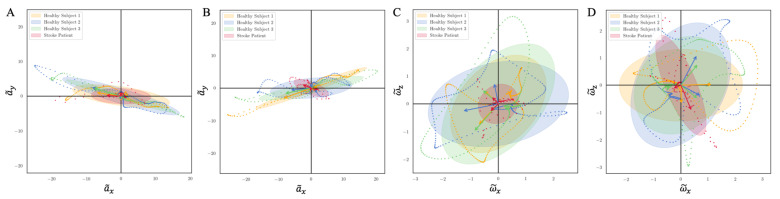
PCA results of $\tilde{A},\;\tilde{W}$ matrices for three healthy subjects and one stroke patient. The dotted lines represent the normalized median cycle of each person. The ellipses show 95% confidence region. The arrows are plotted by the eigenvectors, and its length equal corresponding eigenvalues. (**A**,**C**) For left foot and (**B**,**D**) for right foot.

The PCA results of from three healthy people (yellow, blue, and green) and one stroke patient (red) for the left and right side are shown in Figure 14: A, B denote the PCA results of $\tilde{A}$ metrix, C, D denote the PCA result of $\tilde{W}$ matrix, and A, C and B, D plots are for their left and right side, respectively. In each plot and for each person, the longer arrow denotes the first principal component vector, which indicates the direction of the largest variation in the data. The length of the vector is the corresponding singular value of $\tilde{A}$ or $\tilde{W}$ matrix, which indicates the degree of variation along the direction of the vector. The 95% confidence ellipses based on PCA summarize the clouds of the data points and describe the signal variability containing the underlying mean.

In those plots, the data of the three healthy subjects show different principal components, indicating their personal walking patterns. The data of the stroke patient shows a significant different principal component compared to the healthy subjects. As shown in Figure 14A,B, the singular values of the stroke patient were much smaller than that of healthy subjects. Moreover, the principal component vectors of the stroke patient in Figure 14B even lie in different quadrants from the vectors of the healthy subjects. This agrees with the fact that the right body side of the stroke patient is more severely compromised than the left side. Similar differences can also be observed in Figure 14C,D. The left side (less affected) shows a smaller confidence ellipse, which revealed that the ankle rotation variation of the stroke patient is much smaller than healthy subjects. This also demonstrates that during walking, the patient’s ankle joint is less flexible and the variability of spatiotemporal kinematic joint parameters is much smaller than healthy subjects [38,39]. The right foot (more affected) of the stroke patient demonstrates the largest deviation from normal, with the principal component vector manifesting in a different orientation than in healthy subjects.

These results suggest that the principal components can be a useful metric for quantifying personal gait characteristics and allows deficit detection of abnormal gait when compared to healthy subjects. It proves a valuable metric for tracking gait progress remotely throughout rehabilitation, and will help provide an appropriate treatment during early stages rehabilitation.

## 4. Discussion

In the presented study, we developed a wearable in-shoe gait analysis system embedded with on-board inertial sensors, i.e., accelerometer, gyroscope, and magnetometer, that can transfer data to mobile devices wirelessly via Bluetooth in real-time or store data locally on an SD card without causing inconvenience for the users. The batteries of the shoes are charged wirelessly on a customized docking station, based on electromagnetic induction. This cost-effective, portable, and user-friendly in-shoe system enables real-time gait monitoring even during outdoor settings.

We examined the aliasing effect of an inertial sensor and identified the optimal sampling frequency for walking data acquisition. It is important to identify the frequency distribution of the useful signal information, especially for fast varying signals such as an accelerometer. Inadequate sampling frequency will prohibit capturing high frequency gait signals, and leading to significant drift for spatial gait parameter estimation due to the continuous integration of the sensory data. Despite the advances in the field, this aspect is usually overlooked in the literature. We believe the research in the field will benefit from a standardization effort in determining optimal data acquisition parameters.

To estimate spatiotemporal gait parameters, a well-established ZUPT gait analysis technique was extended with a gradient descent-based Madgwick’s filter and heuristic techniques. The estimation results of spatiotemporal gait parameters compared between the proposed system and the computer-based optical 3DGA system showed a good agreement, with percentage absolute errors of 1.19%, 1.68%, 2.08%, 1.23%, 3.02%, and 2.59% for stride time, stride length, stride velocity, cadence, swing time, and stance time respectively. All p values for those spatiotemporal gait parameters were larger than 0.05, which indicate that there was no significant statistical difference between the two data acquisition system, Nushu and 3DGA. For stride time, stride length, stride velocity, and cadence, the validation results showed small errors. While for swing and stance time estimation, the estimation was relatively worse. Additionally, p values for the asymmetry of swing and stance time was less than 0.05, which indicate that there were significant differences in those parameters between Nushu and 3DGA. This may be due to the reason that swing and stance phases were separated by the transition from foot flexion to dorsal extension. While as the foot is not a rigid body segment, this definition may not hold for all strides and subjects, thus leading to a reduced accuracy of TO detection. Compared to other wearable inertial sensors for healthy subjects’ gait assessment in recent studies, the developed Nushu system demonstrated high accuracy in estimated spatiotemporal gait metrics as shown in Table 4. The estimation error of the stride length and stride velocity showed a relatively small value compared with other wearable sensor-based systems, which indicated that the proposed algorithm is promising for spatial parameter extraction.

The limitation of this study is that we demonstrated our system in a limited small-scale study with healthy subjects. Having validated our hardware and software, we are planning to validate in a larger set of young adults then move to older adults. We are also scheduling clinical studies for the validation of our approach for the diagnostics and monitoring of patient groups (such as Parkinson’s, stroke, and multiple sclerosis) and for the monitoring of specific population groups such as the elderly. Another limitation is that due to the spatial constraints of the reference system, no free-living movements were evaluated in the current study. For different walking conditions and walking speed, the system performance might be affected. The further validation for different walking conditions and walking speed should be conducted, and the validation of the kinematic and kinetic gait parameters should also be considered in the future studies. Besides, incorporating the system with a pressure sensor in future to measure the load distribution across the foot and the center of pressure during standing and walking is valuable for postural stability evaluation [10,43]. Another improvement can be the synchronization of the sensors in left and right shoes with respect to time. In this study, we estimated the spatiotemporal parameters from the left and the right sensors individually without any need for synchronization. However, this can be achieved using a mobile application or a user interface via which multiple sensors can be controlled to start or stop recording simultaneously.

We introduced a new intuitive visualization method of describing spatiotemporal relations between collected gait data using hodographs, which captured clearly the variance in the walking data. It reflected the gait kinematics throughout every single gait cycle, which was different from traditional discrete metrics commonly used in the literature, such as walking speed and stride variability. Moreover, the hodographs clearly displayed important differences in walking characteristics between healthy subjects and stroke patients at the first glance without further processing the gait cycle data, which requires significant effort and introduces inevitable error when applying traditional gait metrics for abnormal gait recognition. Differences in gait patterns can be easily recognizable from graphical hodographs, even with a limited number of gait cycles, e.g., only 12 gait cycles in Figure 12. While different walking subjects and walking speed may have an effect on the graphical walking characteristics, such as slower walking speed leads to greater variabilities, older adults display a greater variability than young adults [44], which leads to a significant different graphical characteristics. Therefore, the effects of different walking conditions on the proposed metric should be investigated in the future studies. In addition, we complemented the visual hodographs with a new quantitative metric provided by a PCA algorithm. In the future, a shape recognition algorithm could further be developed for hodographs in order to perform automatic pathologic gait recognition and classification [45]. The combination of visual and quantitative metrics offers a great potential to become a diagnostic tool for differentiating gait patterns between individuals and tracking the progress of patients during their rehabilitation process.

## 5. Conclusions

In summary, we developed an inertial sensor-based in-shoe system with optimized sampling configurations for gait monitoring and analysis. We demonstrated that our proposed algorithm had a promising performance for spatiotemporal gait parameters estimation. Thus, the developed system has a great potential for clinical and daily life applications. A new gait metric based on graphical eigenanalysis was proposed, and it shows a high potential for gait diagnostic and gait rehabilitation assessment.

## Figures and Tables

**Table 1:** List of gait parameters measured and estimated by the Nushu system.

Gait Parameter	Units	Definition	Reference
Stride Velocity	m/s	Mean in-plane velocity of each gait cycle	[3]
Stride Time	s	Time taken for a full gait cycle	[3]
Stride Length	m	In-plane distance travelled during a gait cycle	[3]
Minimum Foot Clearance	m	Minimum ground clearance of the foot during swing	[1,3]
Strike Angle	deg	Angle of impact w.r.t foot’s mediolateral axis	[1]
Stance Time	s	Time duration from heel strike to toe off	[2,3]
Swing Time	s	Time duration from toe off to heel strike	[2,3]
Stance Phase	%	Ratio of stance time w.r.t gait cycle duration	[2,3]
Swing Phase	%	Ratio of swing time w.r.t gait cycle duration	[2,3,6]
Cadence	strides/min	Number of strides per minute	[2,3]
Maximum Angular Velocity	rad/s	Maximum angular velocity w.r.t the mediolateral axis	[7]
Symmetry	%	Relative difference between left and right feet performance	[7,8,18]
Variability	%	Measure of the walking consistency	[9]

**Table 2:** A summary of subjects’ information.

Subjects	Age	Height (cm)	Weight (kg)
Subject 1	26	165	50
Subject 2	35	165	75
Subject 3	65	162	58
Subject 4	66	168	78

**Table 3:** Accuracy and precision of Nushu estimated spatiotemporal gait parameters.

Gait Parameter	Units	MAE	MAE (%)	RMSE	SD	*p* Value
Stride Time	s	0.012	1.19	0.017	0.011	0.732
Stride Length	m	0.024	1.68	0.030	0.018	0.765
Stride Velocity	m/s	0.029	2.08	0.037	0.025	0.878
Cadence	strides/min	1.390	1.23	1.942	1.389	0.714
Swing Time	s	0.012	3.02	0.015	0.008	0.807
Stance Time	s	0.017	2.59	0.021	0.013	0.684
Swing Phase	%	1.239	3.2	1.502	0.875	0.713
Stance Phase	%	1.303	2.12	1.513	0.792	0.569
SR of Stride Time	-	0.022	-	0.028	0.018	0.607
SR of Stride Length	-	0.021	-	0.028	0.019	0.464
SR of Stride Velocity	-	0.029	-	0.042	0.032	0.509
SR of Swing Time	-	0.047	-	0.058	0.037	**<0.05**
SR of Stance Time	-	0.043	-	0.047	0.021	**<0.05**
Variability of Stride Time	s	0.011	-	0.014	0.009	0.684
Variability of Stride Length	m	0.002	-	0.023	0.012	0.066
Variability if Stride Velocity	m/s	0.002	-	0.029	0.017	0.180
Variability of Swing Time	s	0.012	-	0.018	0.014	**<0.05**
Variability of Stance Time	s	0.014	-	0.016	0.008	0.382

**Table 4:** Accuracy comparison of RMSE (%) and MAE (%) between recent wearable devices and proposed Nushu system.

	Reference	Subjects	Sampling Frequency	Setup	Device Location	Stride Time	Stride Length	Stride Velocity	Cadence
RMSE (%)	Teufl et al. [20].	24 healthy	60 Hz	7 IMUs	Foot, shank, thigh, pelvis,	0.90	2.98	2.72	3.07
	Tunca el al. [30].	22 healthy	100 Hz	2 IMUs	Foot	-	5.2	-	-
	Zhang el al. [40].	9 healthy	500 Hz	2 IMUs, 8 piezoresistive sensors	Foot	-	2.5	2.5	-
	**Nushu**	**4 healthy**	**250 Hz**	**2 IMUs**	**Foot**	**1.60**	**2.06**	**2.71**	**1.71**
MAE (%)	Kluge et al. [22].	11 healthy	102.4 Hz	2 IMUs	Foot	1.1	3.6	3.7	-
	Gonzalez et al. [41].	6 healthy	100 Hz	1 IMUs	L3 vertebra	2	-	-	-
	McCamley et al. [42].	18 healthy	100 Hz	1 IMUs	Spine	2	-	-	-
	**Nushu**	**4 healthy**	**250 Hz**	**2 IMUs**	**Foot**	**1.19**	**1.68**	**2.08**	**1.23**

## Supplementary Materials

The following are available online at https://www.mdpi.com/article/10.3390/s21082869/s1, Video S1: Walking pattern of healthy subject 1. Video S2: Walking pattern of healthy subject 2. Video S3: Walking pattern of healthy subject 3. Video S4: Walking pattern of stroke patient.
