# Supplementary material for: An Intelligent In-Shoe System for Gait Monitoring and Analysis with Optimized Sampling and Real-Time Visualization Capabilities
Source: Sensors (Basel). 2021 Apr 19;21(8):2869. doi: 10.3390/s21082869 (PMC8074136; doi:10.3390/s21082869)
Supplement: Supplementary file 1 [file sensors-21-02869-s001.zip › Supplementary Report.pdf]

magnes

Gait Analysis Report - KISHU

Date:	DD.MM.YYYY
Subject:	Name OR Identifier
Test ID:	#
Height:	X.XX m
Weight:	YYY.YY kg

Jiaen perfomed a gait test with the gait analysis system KISHU. The reported values represent the average behaviour for a total of 23 steps.

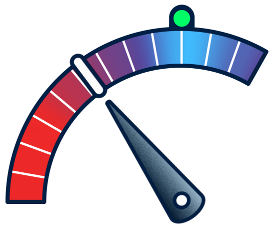

Gait speed

Gait speed represents the average speed of each step, which corresponds also to the overall average walking speed during the trial. The mean gait speed has been 0.77m/s, which is considered to be low.

The The fall risk associated with the measured gait speed is not given.

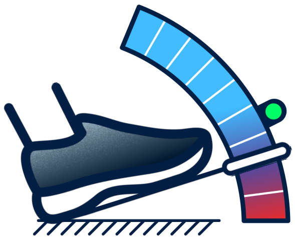

Strike angle

The heel strike angle represents the average angle at which the foot hits the floor.

The The average strike angle has been 17.5 degrees, which is considered to be below-average.

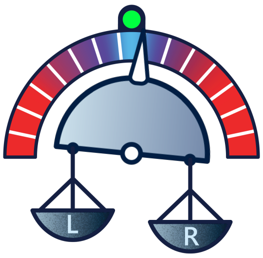

Symmetry

Parameters as stride length, swing velocity and strike angles of both left and right foot contribute to assess symmetry, which is given as how much work each foot does compared to the optimum (50%-50%). The measured symmetry has been 48.6%-51.4% (respectively for the left and right foot), which is to be considered average.

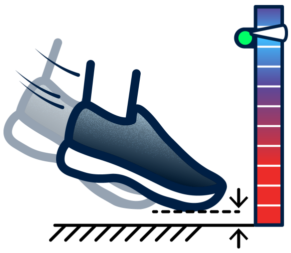

Minimum toe clearance

Minimum toe clearance (MTC) is the average minimum distance from the floor which occurs during the swing phase at each walk cycle. The MTC measured is 1.6cm, which is to be considered average.

magnes

Stance and Swing phases

Stance phase and swing phase are measured by considering the average duration of the swing and stance phases for each walking cycle. Stance and swing phases have been 63.3% and 37.0% respectively, which are associated with a slightly below-average pace.

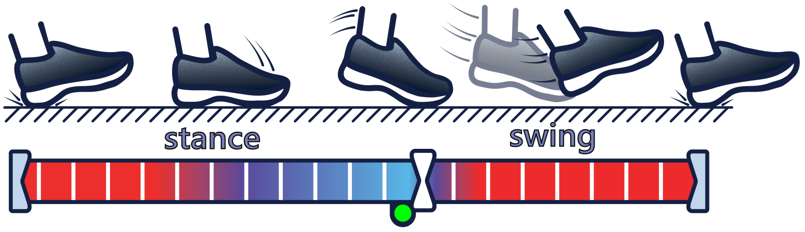

Stride length

Stride length is the average distance travelled by the same foot at the end of each walk cycle. The average stride length has been 1.06m, which is considered to be slightly below average.
The The fall risk associated with the measured stride length is not given.

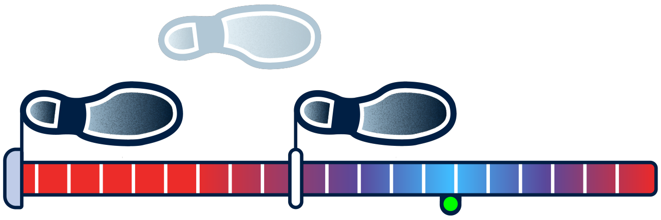

Detailed overview

This table gives a overview about both feet's average parameters and the measured variability. Variability is a measure of how inconsisent is someone's walking pattern and it's represented by a percentage of the mean deviation with respect to the average value.

	Left	Var [%]	Right	Var [%]	Goal	Var [%]
Gait Speed[m/s]	0.81	15.6	0.73	45.2	1.3	-
Strike Angle[°]	19.33	20.7	15.65	40.9	27	-
Sup Angle[°]	-2.1	-308.5	12.52	46.8	-	-
MTC[cm]	1.25	0.4	1.96	0.1	1.55	-
Stance phase[%]	63.77	15.4	62.89	14.7	60	-
Stride length[m]	1.12	10.2	0.99	42.7	1.65	-
Max ang. vel.[°/s]	5.14	10.1	5.07	17.4	-	-
Cadence[steps/min]	42.97	7.6	42.69	7.7	-	-
